# Quantitative laryngoscopy with computer-aided diagnostic system for laryngeal lesions

**DOI:** 10.1038/s41598-021-89680-9

**Published:** 2021-05-12

**Authors:** Chung Feng Jeffrey Kuo, Wen-Sen Lai, Jagadish Barman, Shao-Cheng Liu

**Affiliations:** 1 Department of Materials Science and Engineering, National Taiwan University of Science and Technology, Taipei, Taiwan, Republic of China; 2 Department of Otolaryngology-Head and Neck Surgery, Taichung Armed Forces General Hospital, Taichung, Taiwan, Republic of China; 3 Department of Otolaryngology-Head and Neck Surgery, Tri-Service General Hospital, National Defense Medical Center Taipei, No. 325, Sec. 2, Cheng-Gong Road, Neihu District, Taipei, 114 Taiwan, Republic of China

**Keywords:** Computational science, Information technology, Anatomy

## Abstract

Laryngoscopes are widely used in the clinical diagnosis of laryngeal lesions, but such diagnosis relies heavily on the physician's subjective experience. The purpose of this study was to develop a computer-aided diagnostic system for the detection of laryngeal lesions based on objective criteria. This study used the distinct features of the image contour to find the clearest image in the laryngoscopic video. First to reduce the illumination problem caused by the laryngoscope lens, which could not fix the position of the light source, this study proposed image compensation to provide the image with a consistent brightness range for better performance. Second, we also proposed a method to automatically screen clear images from laryngoscopic film. Third, we used ACM to segment automatically them based on structural features of the pharynx and larynx, using hue and geometric analysis in the vocal cords and other zones. Finally, the support vector machine was used to classify laryngeal lesions based on a decision tree. This study evaluated the performance of the proposed system by assessing the laryngeal images of 284 patients. The accuracy of the detection for vocal cord polyps, cysts, leukoplakia, tumors, and healthy vocal cords were 93.15%, 95.16%, 100%, 96.42%, and 100%, respectively. The cross-validation accuracy for the five classes were 93.1%, 94.95%, 99.4%, 96.01% and 100%, respectively, and the average test accuracy for the laryngeal lesions was 93.33%. Our results showed that it was feasible to take the hue and geometric features of the larynx as signs to identify laryngeal lesions and that they could effectively assist physicians in diagnosing laryngeal lesions.

## Introduction

Structural abnormalities in the larynx or inconsistencies in neuromuscular coordination may cause an abnormal sound. About 90% of such abnormal sounds are caused by structural voice disorder^1^, including vocal cord polyps, cysts, leukoplakia, and tumors. Laryngoscopic examination may result in disagreement in diagnosis among providers^2^. To assist physicians in the correct diagnosis and appropriate treatment for patients, computer analysis of endoscopic images has been used in many studies to objectively diagnose laryngeal lesions. Menon et al.^3^ proposed using the glottic contour as the edge of the vocal cords to carry out optical flow analysis for statistical motion vectors on the left and right vocal cords respectively, and proved that the normal left and right vocal cords had similar motion vector statistics, which could effectively distinguish vocal cord paralysis and normal vocal cords. However, the analysis of vocal cord-based patterns without assessing other information can be applied only to neurogenic or functional voice disorders. Mendez et al.^4^ used image segmentation technique i.e. active contour method (ACM) to detect the morphological pathologies by using color images in RGB format, and then used connected component analysis to select the region of interest (ROI) as central component of foreground class for features measurement. The sizes of the polyps or cysts and the glottal spaces and locations were collected into a database to provide complete pathology information that would help doctors diagnose vocal cord disease. However, the literature does not mention how to correct the lens position, and the distance between the endoscopic lens and vocal cords is not consistent, thereby making it difficult to objectively compare the features of polyps and cysts. Verikas et al.^5^ used a computer-aided diagnosis system to automatically analyze vocal cord images for disease classification. Based on texture, hue, and geometric features, the pattern classifier was applied to categorize images into healthy, diffuse corditis, and nodules, with an accuracy of 87%. However, this system still has its limits in detecting multiple lesions simultaneously.

Previous literatures have used static images for an automated analysis to categorize diseases of vocal cords. In this study, for the first time we take image automatically from endoscope video for the analysis. The clearest image was selected automatically for segmentation. By using hue and geometric analysis for the laryngeal region, automatic pathological change detection and classification were proposed for healthy vocal cords and laryngeal lesions, including vocal cord polyps, cysts, leukoplakia, and tumors. Image processing technology was applied to quantify the changes in the larynx caused by specific lesions. So, the doctor could support his diagnosis with our system results.

## Materials and method

Laryngoscopic images were provided by a tertiary referral center. Our clinical database included a Total of 284 patients. Among them, 38 samples of healthy vocal cords and 246 samples of vocal cord lesions were collected, including 76 vocal polyps, 62 vocal cysts, 52 vocal leukoplakia and 56 vocal tumors. Where training samples were 200, validation samples were 74 and testing samples were 10. In the validation samples healthy, vocal cord polyps, cysts, leukoplakia, and tumors were 14, 18, 16, 12 and 14, respectively. Patients were excluded from this study if they had anemia, smoking habit, asthma, and those who had previously received radiotherapy or surgery in head and neck region.

### Image processing

Problems like uneven brightness distribution and low contrast in laryngoscopic images could lead to inaccurate segmentation and inconsistent results. In this study, contrast-limited adaptive histogram equalization (CLAHE) was used to reduce these problems^6–8^. First, adaptive histogram equalization (AHE) with good local contrast was used to divide the images into different grid regions, which limited the histogram to a small region so as to perform operations via different regions. The grayscale values of the upper and lower limits were expanded from 0 to 255 by contrast enhancement of the maximum and minimum gray values of the region; if the region was large, the contrast would be reduced, and vice versa. Later, CLAHE was used to effectively solve the additional problem of noise amplification due to the contrast adjustment. To smooth and remove the noise resulting from swaying, swallowing, or saliva reflecting light from laryngoscopic images, Gauss smoothing was used to remove noise by calculating weighted average of each pixel’s neighborhood where neighborhood pixel’s value becomes or lower closer to the central pixel, improve the areas where the gray scale values changed dramatically, and reduce the segmentation errors in subsequent steps.

The grayscale value was used to automatically segment the glottis. In order to highlight the difference in image brightness and retain features details of ROI pixel’s during segmentation, and avoid the result of over-segmentation, the fast Otsu method^9^, which is less computational, was used to overcome the disadvantage of the traditional Otsu method. By calculating the average grayscale value of the entire image (icp_m_), images above the average grayscale value were regarded as the foreground (icp_f_), and vice versa as the background (icp_b_). The search area was divided into 0 ~ icp_b_, icp_b_ ~ icp_m_, icp_m_ ~ icp_f_ and icp_f_ ~ 255. The inter-group variances of the four regions were calculated separately, and the largest inter-group variance was regarded as the possible optimal threshold. For the subsequent vocal cord segmentation, we used the ACM^10^ as the image segmentation method. The vocal cords and the surrounding tissue boundaries were blurred. The algorithm defined the initial parameterized curve, and made the curve move to the target boundary by minimizing the energy function, i.e. the reconciliation of the internal and external forces of the image. The initial parameterized curve was mutually pulled by the internal and external forces of the energy function, and finally the balance of the forces, that is, the minimized energy function, was achieved, and the final contour boundary was obtained. Details on CLAHE, the fast Otsu method and ACM processing, and the parameters of the Gaussian template are provided in the Supplementary section.

### Classifier

In this study, the extracted histogram of the segmented image were classified using a support vector machine (SVM), which was able to find the optimal demarcation hyperplane in limited samples[^11, 12^]. Its functional design is to maximize boundaries between the classes and its main purpose is to maximize the hyper-plane. The SVM solves the quadratic problems repeatedly during the training. After maximize the boundaries and the hyper-plane, it can effectively classify classes. The purpose of this study was to identify healthy vocal folds and the different types of laryngeal lesions, including vocal cord polyps, cysts, leukoplakia, and tumors. The decision tree method was used to extend the SVM from binary classification to multivariate classification required for this study, and the SVM was used to classify data points with unclear decision boundaries. The decision tree was implemented to perform the non-linearly separable data points to quickly classify laryngeal lesions, which can reduce the training time of the test set.

### Image screening

**Figure 1:**
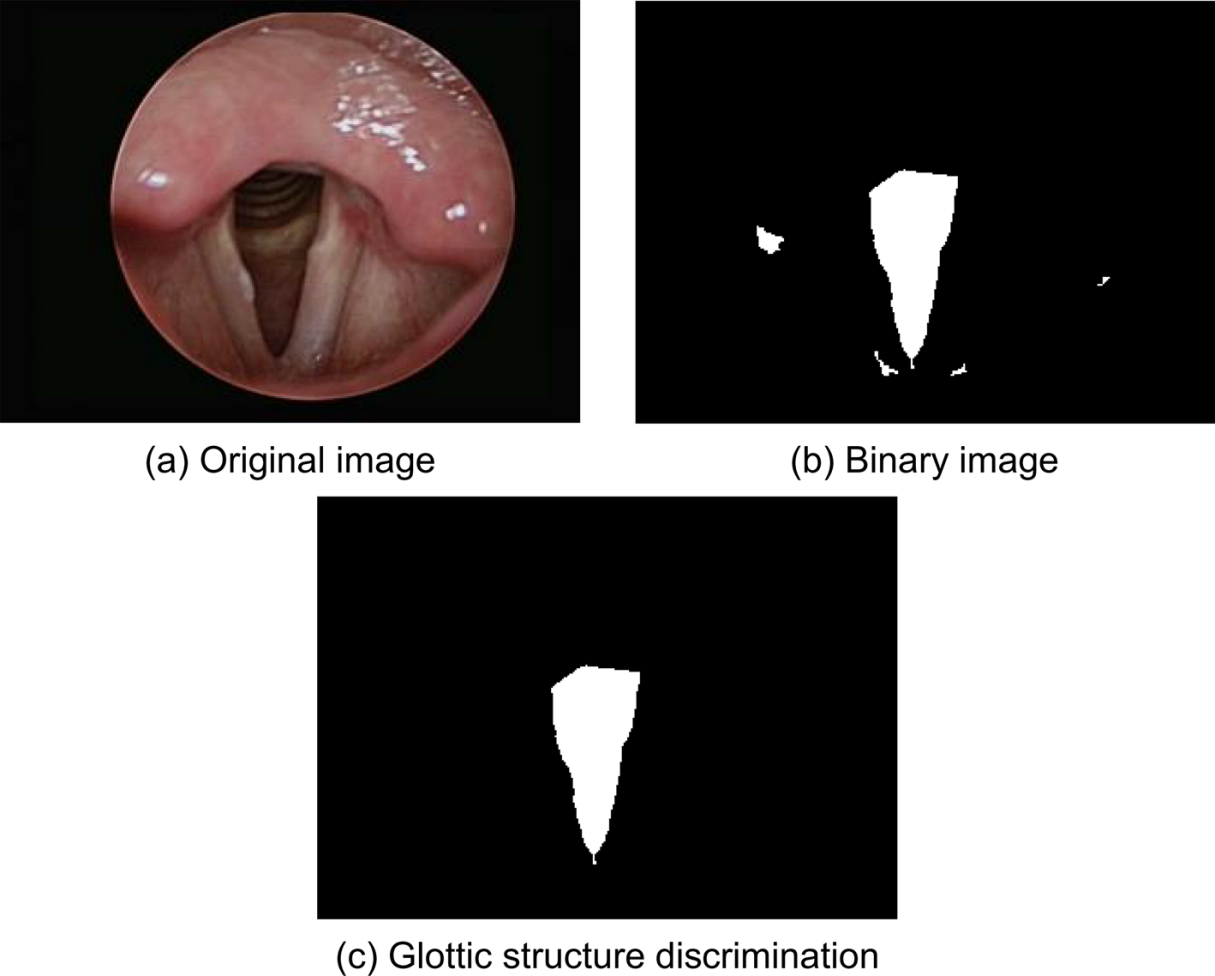
Glottic segmentation process.

The clearest images from the dynamic throat images were searched automatically for following segmentation and feature analysis. The algorithm performs the noise identification and used the Peak Signal to Noise Ratio (PSNR) to calculate error between each frame in the video. Therefore, if error is very low than it can automatically accept the frame or the clearest image. The automatic segmentation area included the glottis, left vocal cords and right vocal cords. The hue and geometric features of each part were analyzed. The detailed process is as shown in supplementary Fig. 1. The glottal structure varies due to individual based on age, sex and gender, so this study used glottal structure conditions for screening, as shown in Fig. 1. The conditions are as follows:

(1). Centric position: The vocal cords can be observed only when the glottis is at the center of the image, so the areas with high and low glottis are excluded. In this study, the Y axis in the centroid position and if the pixel values are below 50 or above 240 was filtered out.

(2). Aspect ratio: The shape of the glottis is an inverted triangle. The aspect ratio method can be used to filter out the area towards the east–west direction. In this study, the area with an aspect ratio greater than 0.85 was filtered out.

(3). Area size: The area refers to the sum of the pixels in the area. This study excluded blocks with an area of less than 900 pixels. The purpose was to remove the excess area other than the glottis.

**Figure 2:**
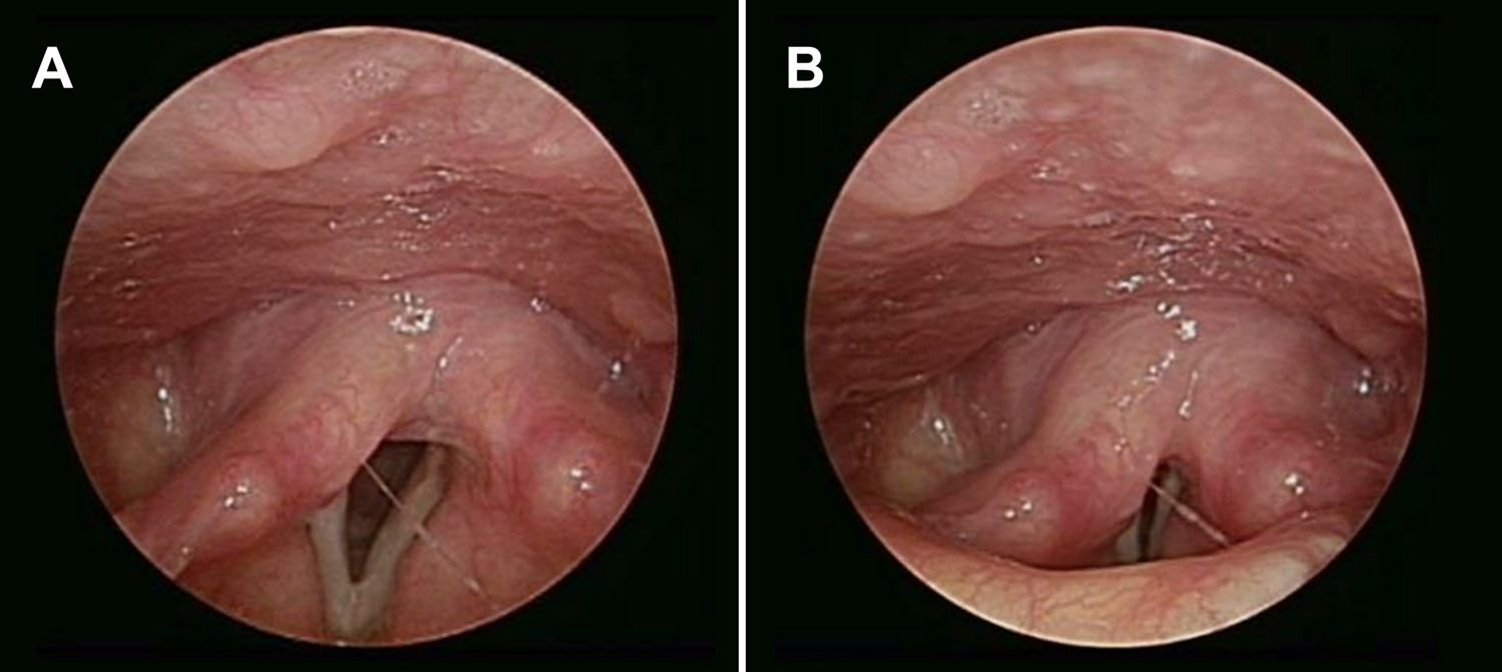
Filter clear vocal cord images. (**A**) The glottis area is the largest, and the vocal cord image is clear. (**B**) The glottal area is too small, and the vocal cord image is blurred.

For subsequent image segmentation, it was necessary to select laryngeal images with a consistent standard from the laryngeal endoscopic video. The number of glottic block pixels of all laryngeal images with glottis was counted in this study. Finally, the largest glottal block in each image was retained as the laryngeal image for subsequent segmentation, because the vocal cord image could be clearly identified, as shown in Fig. 2. The peripheral part of the laryngeal image (black part) was the redundant part during calculation; it was unified to the grayscale pixel value of 0 for subsequent calculation, as shown in supplementary Fig. 2.

### Image compensation

**Figure 3:**
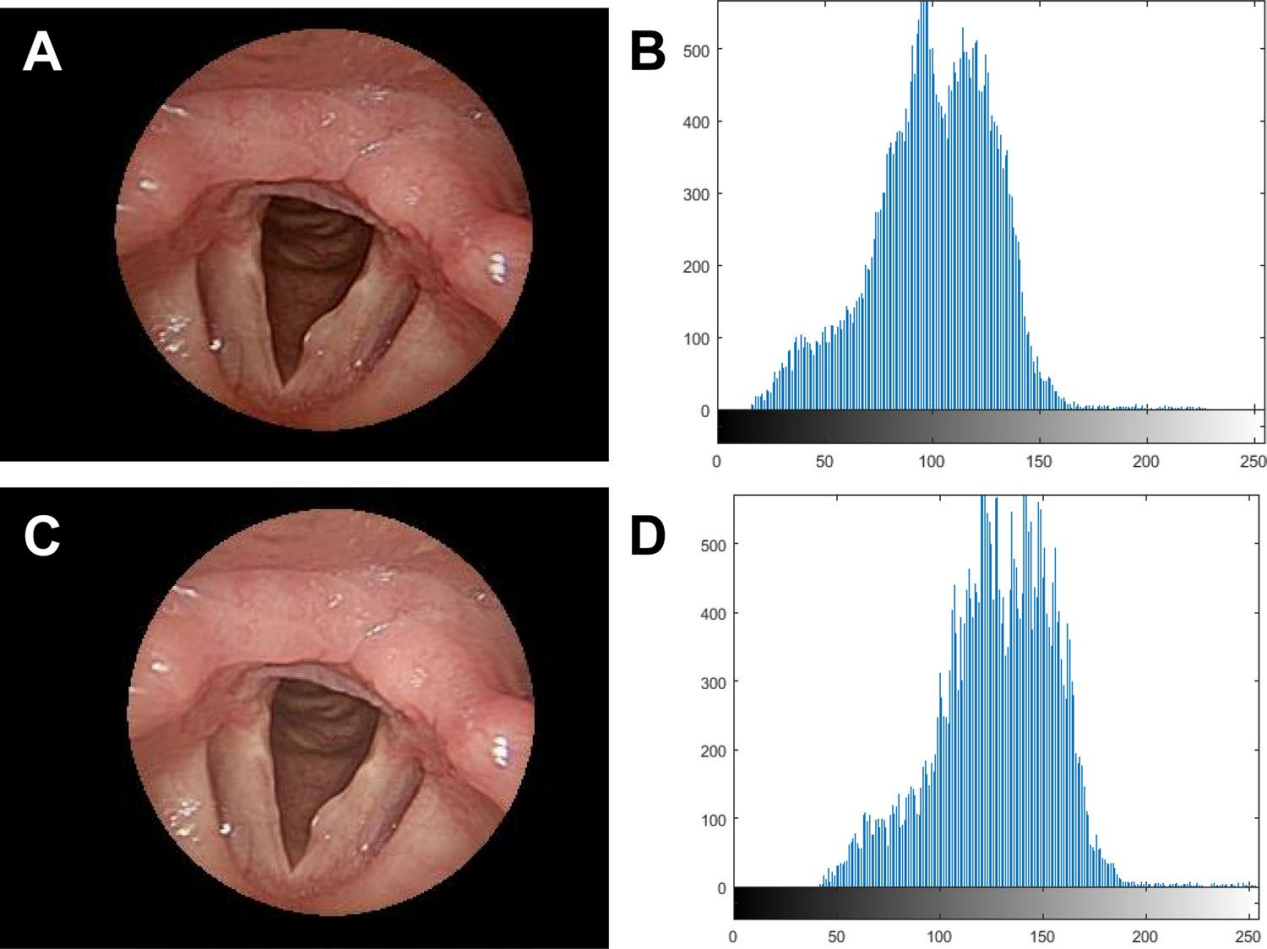
Image compensation. (**A**) Original image. (**B**) Original image brightness histogram (average value: 100.64). (**C**) Image compensation. (**D**) Image compensation brightness histogram (average value: 125).

When taking pictures of the larynx, the physiological reaction of the patient often made the laryngoscopic images unable to be fixed at the shooting position, resulting in inconsistent illumination. For example, when the lens position was far away, the illumination was darker, and vice versa. To ensure the images have similar color ranges, this study used the histogram translation method to compensate for the difference in the current average value for each pixel, making each laryngeal area of the image reach a similar gray scale value. The gray scale characteristics of each region of the image were clearly demarcated, so as to make sure the subsequent image segmentation and feature analysis had better results and credibility. Figure 3 presents an image before and after translation. The conversion formula is shown in Eq.:

$$I\left( {x,y} \right) = O\left( {x,y} \right) + \left( {S - O_{m} } \right)$$

where *x*, *y* is the image coordinate position, *I* s the grayscale value of the image after translation, *O* is the grayscale value of the current image, *S* is the set value (here the set value is 125 to ensure not to over compensate), and *O*_*m*_ is the average value of the current image.

### Area segmentation

The glottic area was segmented in the previous step, and then the left and right vocal cords were segmented. The segmented areas were removed from the original image to avoid subsequent segmentation errors. In this study, the vocal cords were segmented and its edge image was extracted. The glottis was transected along the glottic centroid, and then the lowest point of the glottis was searched to vertically cut the lowest point. The image left was the seed image of the vocal cords, as shown in supplementary Fig. 3. Based on the seed image, it will find the edge of the glottis. We used ACM to segmented the glottis and this ACM method applied to different glottis geometric location so that edges can be extracted for lesion classification.

**Figure 4:**
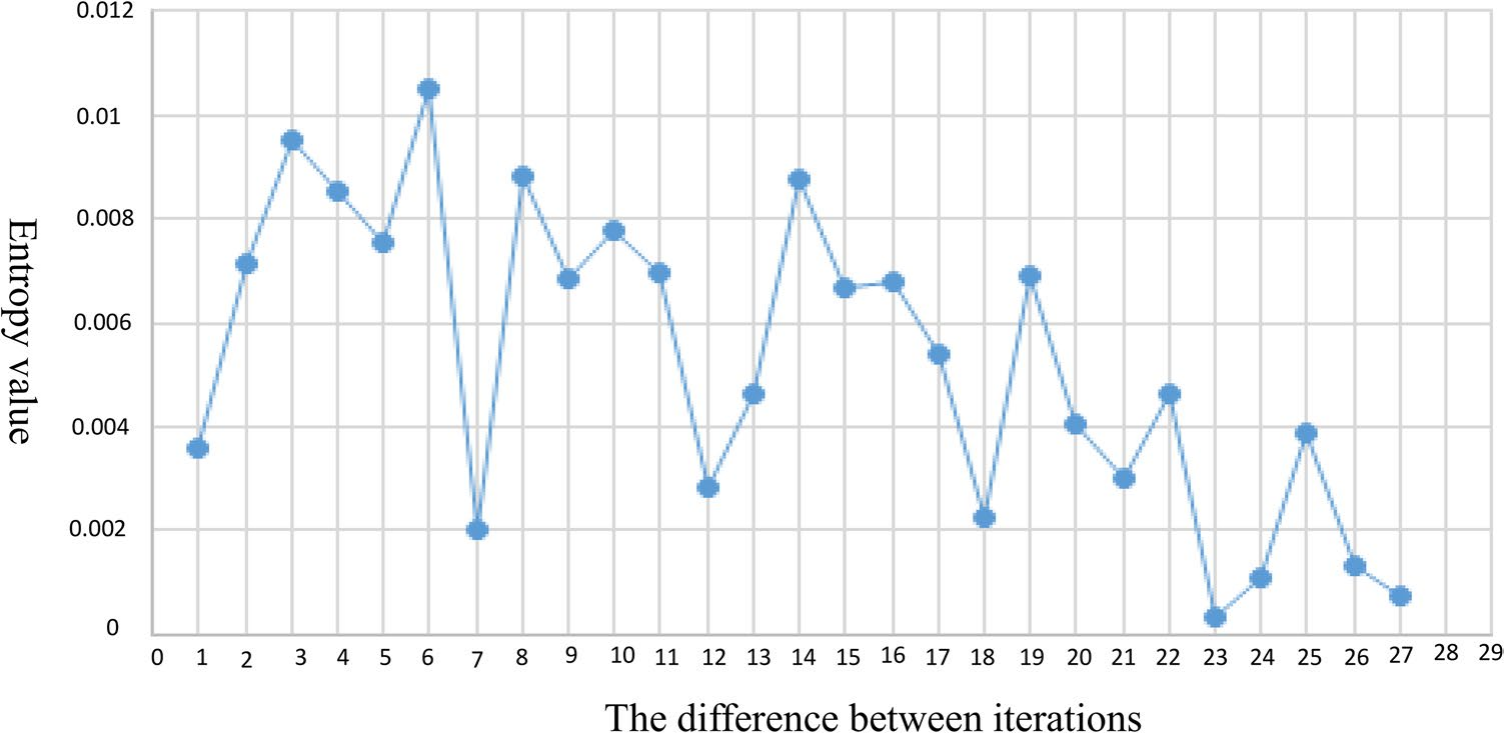
Entropy difference between iterations.

**Figure 5:**
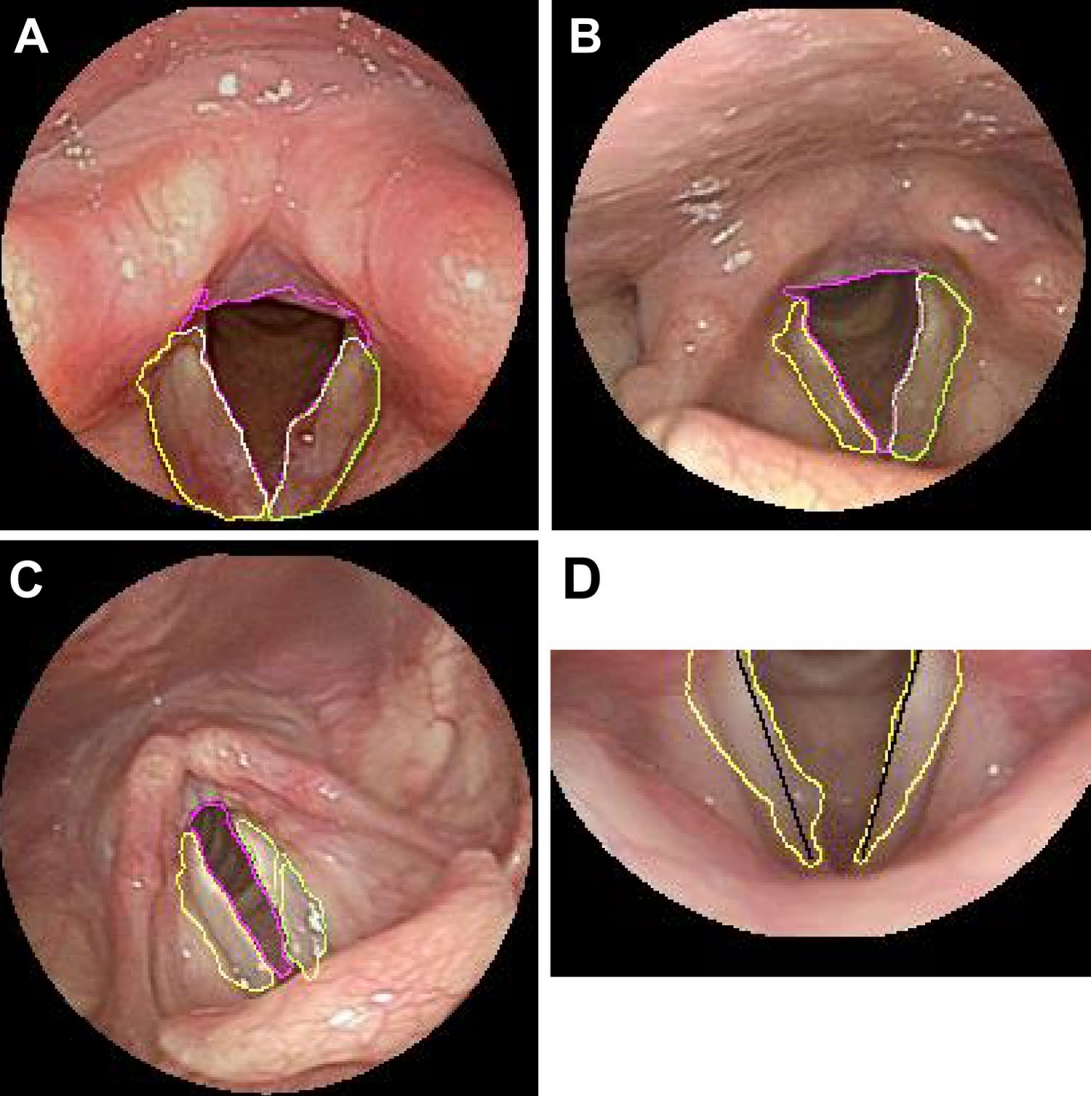
Adaptive vocal cord segmentation. (**A**) The number of iterations of the right and left vocal cords was 38 and 35, respectively. (**B**) The number of iterations of the right and left vocal cords was 5 and 18, respectively. (**C**) The number of iterations of the right and left vocal cords was 22 and 20, respectively. (**D**) Vocal image (yellow) and two straight lines (black) were used to calculate the geometric features.

Since the size of the vocal cord in the image varies greatly because of the lens distance, with close distances producing a magnified effect, the number of iterations using the ACM cannot be segmented by fixed values. To solve this problem, this study proposed an adaptive way to determine the number of iterations, that is, the gap between the vocal cords and pseudo-vocal cords can make the growth range of the active contour method relax gradually, so it can be used as the basis to distinguish the two. Therefore, for each iteration, the entropy of the growing range was calculated and subtracted from the entropy of the growing range in the previous iteration to find the iteration number with minimum entropy difference, which gave the optimal number of iterations of the vocal cords. In this study, the minimum number of iterations given by the ACM was 13 and the maximum was 41. The entropy of the growth range of each iteration was calculated, and the difference in entropies between iterations was calculated to find the minimum difference, as shown in Fig. 4. In this case, the minimum entropy difference was 23 times, and the entropy difference was 0.0003. Because the minimum number of iterations by the ACM in this study was 13 and the minimum difference of entropy was 23—which means that the growth range of the ACM in the 35th iteration and 36th iteration had reached saturation, with the optimum number of iterations in this case being 35—we were able to find the optimum number of iterations of the left and right vocal bands, as shown in Fig. 5. To determine the segmentation accuracy of each sample in the experiment, the numerical value obtained by manual selection was taken as the real value, and the value selected by the program automatically was taken as the measurement value. The relative error was used for analysis.

### Feature analysis

Vocal cord polyps and cysts are convex objects on the vocal cords. Geometric features are used to identify whether the vocal cords have abnormal appearance. In this study, vocal cord geometry was used to identify whether there were convex objects on the vocal cords. A straight line was drawn along the vocal fold. If an object had vocal cord line beyond protruding lines, the geometric feature was judged as abnormal, as shown in Fig. 5D, where the geometry of the right vocal cord (yellow line) has been adjudged abnormal since its protrusion beyond the straight line (black) is pronounced. If there are abnormalities, then the length-to-width ratio of protrusions would be analyzed with the purpose of distinguishing polyps from cysts (details are provided in the Supplementary section). In addition to protrusion, the vocal tumors were identified with their hue feature, which showed different severity from the hue feature of leukoplakia. In order to detect hue changes of the vocal cords, the standard deviation of the grayscale value in the vocal cord area was used as the standard. When the standard deviation was too large, the grayscale value of the image changed drastically, that is, the vocal cord hue changed. The difference between leukoplakia and tumor was identified and analyzed.

### Ethical considerations

The research protocol (No: 1-108-05-132) was reviewed and approved by the Institutional Review Board of the Tri-Service General Hospital, Taipei, Taiwan. All methods were performed in accordance with the relevant guidelines and regulations. All patients provided written informed consents prior to participation.

### Consent for publication

Consent to publish has been obtained from all participants.

## Results and verification

### Adaptive vocal cord segmentation

In this study, 10 samples were randomly selected and taken as testing purposes as well as for comparing segmentation accuracy. Others were used to training and rest if they were used for training and validation. Manual segmentation by three physicians was compared with the method proposed in this study, and the comparison results are shown in Table 1; these results gave the average relative errors between the three physicians’ method and the proposed method. The average relative error for the left vocal cord was 4.35%, the average relative error for the right vocal cord was 5.73%, and the average relative error for the glottis was 3.08%. The proposed automatic segmentation was verified to be reliable.

**Table 1:** Average relative error of three parts of ten samples.

Sample	Region		
	Left vocal cord (%)	Right vocal cord (%)	Glottis (%)
No. 1	0.17	6.89	4.22
No. 2	3.19	8.35	2.91
No. 3	5.10	7.93	3.55
No. 4	5.15	5.08	3.94
No. 5	3.99	5.05	2.16
No. 6	4.16	6.89	2.95
No. 7	5.18	4.57	4.30
No. 8	3.12	3.89	1.92
No. 9	6.69	5.83	2.94
No. 10	6.84	2.90	1.93
Average relative error	4.35	5.73	3.08%

### Analysis of vocal cord lesion features

**Figure 6:**
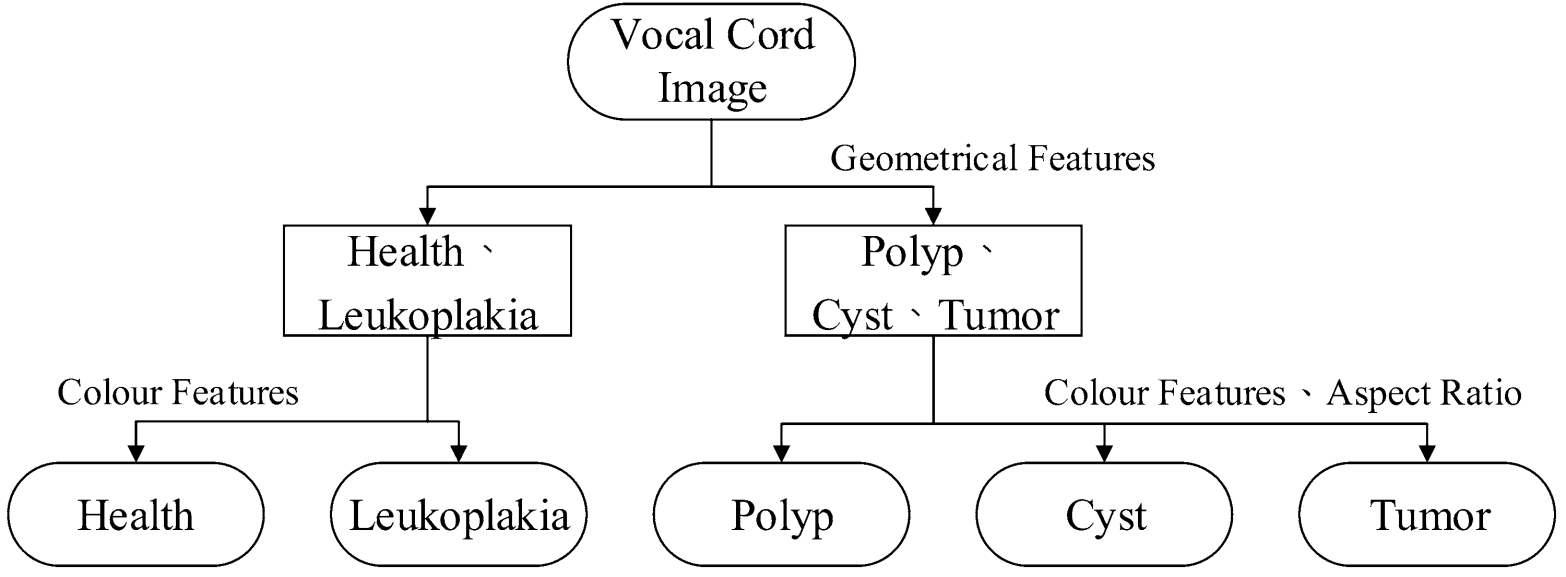
Lesion classification model.

By analyzing the features of the laryngeal image using an image processing technology, we were able to discriminate healthy vocal cords from laryngeal lesions, including vocal cord polyps, leukoplakia, cysts, and tumors, in real time. Firstly, training samples were used to construct the SVM model, and then the test samples were classified based on the trained SVM model, as shown in Fig. 6.

In this study, 10 hierarchical cross-validation verifications were used to evaluate the classification results. The overall recognition rate was 97.56%, and the identification results are shown in Table 2. The recognition rate of laryngeal lesions by the SVM was 93.15% for polyps, 95.16% for cysts, 100% for leukoplakia, and 96.42% for tumors; the recognition rate for healthy cords was 100%. Whereas the cross-validation accuracy for the five classes were 93.1%, 94.95%, 99.4%, 96.01% and 100%, respectively. The overall recognition rate was 96.47%. The SVM represents high distinction, high independence and high reliability. The classifier selected could effectively identify laryngeal diseases because of decision tree based SVM classifier. In decision tree based SVM classifier helps the model to work on non-linear data points.

**Table 2:** Vocal cord lesion identification results.

Lesion	Sample				
	Polyp (76)	Cyst (62)	Leukoplakia (52)	Tumor (56)	Healthy (38)
Training samples	55	43	38	40	22
Recognition rate	93.15%	95.16%	100%	96.42%	100%
Validation samples	18	16	12	14	14
Recognition rate	93.1%	94.95%	99.4%	96.01%	100%
Testing samples	3	3	2	2	2
Recognition rate	66.66%	100%	100%	100%	100%

## Discussion

To diagnose laryngeal diseases, physicians use laryngoscopy to evaluate the color, shape, geometry, irregularity, and roughness of vocal cords. The results of the examination are subjective and depend upon the physicians’ own experience. Verikas et al.^5^ used vocal cord images to analyze hue, texture, and geometric features, and classified the images, according to their patterns, into three categories: healthy, nodular, and diffuse. However, it is difficult to detect multiple lesions simultaneously by pattern classification. The current study used the decision tree-based support vector machine to detect vocal cord lesions, improving the classification speed of the SVM in the test phase. Based on the test results presented in Table 2, the overall vocal cord lesion recognition rate was 93.33%. The geometric features of vocal cords can be used to identify abnormal vocal cords. Although this study used differences in appearance, there were occasional misjudgments in the identification of polyps and cysts. This was not surprising, as the differences between the two are mainly diagnosed by means of histopathological microscopy. The change in hue caused by vocal cord leukoplakia varies from person to person. This study effectively distinguished the degree of color change in vocal cords using the standard deviation of grayscale values in the vocal cord area. Even when the light source was different or different endoscopes and light source machines were used, the algorithm could be adjusted to a uniform standard for interpretation and identification. Tumors exhibit changes in both hue and texture, and these two features were effectively used to distinguish such lesions.

A common problem in the field of laryngoscopic image recognition is that it is difficult to capture clear images, and the inability to fix the lens in position results in inconsistent illumination. Many studies have used video recording to analyze glottic motion in order to detect laryngeal lesions^13–15^; however, the literature rarely mentions how laryngoscopic images standardize light sources and how to obtain clear images. Based on the characteristic of the clear image contour, to remove blurred images during image screening, it has been proposed to find the clearest laryngeal image in the laryngeal endoscopic film using the largest area of the glottis. In this study, the proposed image compensation method used the brightness translation method to give different laryngoscopic images a consistent brightness range, and pulled the average brightness of all samples to the grayscale value of 125, to ensure that the images would not be over-compensated. Despite the use of different endoscopes or light sources, effective and standard analysis can be performed according to this method, and the results were valid, consistent, and reproducible.

In this study, the three parts used for the automatic segmentation of the larynx were the glottic space and the left and right vocal cords. The purpose of the glottis segmentation was to screen images, and for this segmentation to act as seed points for the vocal cord segmentation, therefore no follow-up feature analysis was needed for the glottic region. Because a laryngoscopic image cannot fix the shooting distance, the vocal cord size will vary with the lens distance. For example, a vocal cord image will be larger when the lens is closer but smaller when the lens is far, resulting in numerous iterations of the ACM without a fixed value. Some studies have demonstrated that color and texture analysis may be used to classify images in patients with reflux laryngitis. The brightness and saturation could also provide additional inputs for analysis, but these parameters are dependent on the lighting and positioning of the laryngoscope^16^. In this study, adaptive vocal cord segmentation and automatic searching of the optimal number of vocal cord iterations were proposed to solve the problems caused by the lens position and provide good segmentation accuracy. Segmentation will not be accurate if the sizes of the left and right vocal cords are inconsistent. This study verified that the error values of the left vocal cord, right vocal cord, and glottis segmentation were 4.35%, 5.73%, and 3.08%, respectively.

In this study, image processing technology was used to realize the hue and geometric quantitative data of laryngoscopic images, which differed from the subjective clinical observations of physicians and was helpful for the objective analysis of laryngeal lesions. The methods of laryngeal endoscopic film automatic screening of clear images and image compensation proposed in this study could automatically segment areas and analyze their hue and geometric features using laryngeal endoscopic film or a single laryngoscopic image. The proposed method takes only 0.1 s for performing the segmentation and classification of each CT image. In this study, laryngeal lesion identification had high classification accuracy.

## Conclusion

As it is difficult to take clear images by laryngoscopy, this study proposed searching for the clearest of the images taken by a laryngoscope, so as to solve the problem of difficulty of capturing clear images. To eliminate the light source problem caused by inconsistent lens positions of the laryngoscope, the histogram translation method was used to give all samples a consistent grayscale range and subsequently facilitate the part segmentation and feature analysis. Automatic laryngeal segmentation reduced the subjective difference found in manual segmentation and saved time. The proposed decision tree-based support vector machine algorithm had the advantages of fast classification and high classification accuracy. Using three distinctive features of laryngeal lesions with the SVM, the detection results for laryngeal lesions had high accuracy and could assist with relevant medical decision-making.

## Supplementary Information

Supplementary Information.
