# Supplementary material for: Quantitative laryngoscopy with computer-aided diagnostic system for laryngeal lesions
Source: Sci Rep. 2021 May 12;11:10147. doi: 10.1038/s41598-021-89680-9 (PMC8115147; doi:10.1038/s41598-021-89680-9)
Supplement: Supplementary file 1 — Supplementary Information. [file 41598_2021_89680_MOESM1_ESM.doc]

**Supplement:**

Title: Quantitative laryngoscopy with computer-aided diagnostic system for laryngeal lesions

Chung Feng Jeffrey Kuo^1#^, Wen-Sen Lai^2#^, Shao-Cheng Liu^3*^

*^1^Department of Materials Science & Engineering, National Taiwan University of Science and Technology, Taipei, Taiwan, Republic of China*

*^2^Department of Otolaryngology-Head and Neck Surgery, Taichung Armed Forces General Hospital, Taichung, Taiwan, Republic of China*

*^3^Department of Otolaryngology-Head and Neck Surgery Tri-Service General Hospital, National Defense Medical Center Taipei, Taiwan, Republic of China*

**^#^**Co-first authors

**^＊^**Corresponding author:

Shao-Cheng Liu, M.D. PhD.

Associate Professor

Department of Otolaryngology-Head and Neck Surgery,

Tri-Service General Hospital, National Defense Medical Center,

No. 325, Sec. 2, Cheng-Gong Road, Neihu District, Taipei, Taiwan 114, R.O.C.

Tel: 886-2-8792-7192

Fax: 886-2-8792-7193

E-mail: [m871435@ndmctsgh.edu.tw](mailto:m871435@ndmctsgh.edu.tw)

**Running title:** Computer-aided quantitative laryngoscopy

**Author contribution:**

1. **Chung-Feng Jeffrey Kuo,** PhD: study design, critical article review/editing.

2. **Wen-Sen Lai**: acquisition of data, data analysis and interpretation, article and images review/editing.

3. **Shao-Cheng Liu**, MD, PhD: study design, data collection, literature search, images editing, article drafting, article submission.

**Supplement:**

***Contrast-limited adaptive histogram equalization (CLAHE)***

There exist some problems such as uneven brightness distribution or low contrast in laryngoscope images, which will lead to inaccurate segmentation and inconsistent eigenvalues. In this study, CLAHE was used to improve [6-8].

First, a global histogram equalization was performed on an image of the laryngoscope, resulting in the loss of details in areas where the images were too bright or too dark. Therefore, an adaptive histogram equalization (AHE) with good local contrast was used to divide the images into different grid regions, which limited the histogram to a small region so as to perform operations via different regions.

Expanding the grayscale values of the upper and lower limits from 0 to 255 by contrast enhancement of the maximum and minimum gray values of the region; if the region was large, the contrast would be reduced, the area was small, and the contrast would be enhanced, which can improve the situations of overall strong or dull image contrast, or partial loss of details. When the pixels contained in the region were very similar, the histogram would be very concentrated, causing a very narrow range of pixels to be mapped to the entire pixel range, followed by amplifying the noise of the local region at the same time. To solve the problem of noise amplification, contrast-limited adaptive histogram equalization (CLAHE) used the contrast limited method to equally distribute the histogram above the set height to the low-end histogram, thereby reducing the slope of the cumulative distribution function, and the higher the threshold setting was, the higher the contrast would be. Later, AHE could be used to effectively solve the additional problem of noise amplification due to the contrast adjustment.

***Image smoothing***

Swaying or swallowing, saliva reflecting light of laryngoscope images leads to images noises, which needs to be smoothed to remove noise. In this study, Gauss smoothing was used to change the core parameters, improve the areas where the gray scale values change dramatically, such as saliva reflecting light etc., to reduce the segmentation errors in subsequent steps. Gaussian smoothing increased the weight of the pixel near the center, which reduced the fuzzy phenomenon after processing. The parameters of the Gaussian template were calculated by the Gaussian function, and the current pixel values were replaced by adding the result of each pixel in the template, and then the convolution was used to process the entire image. The Gaussian function is shown as Eq. (1)

| $h(x,y)=\frac{1}{2\pi\sigma^{2}}e^{-\frac{x^{2}+y^{2}}{2\sigma^{2}}}$ | (1) |
| --- | --- |

where $\sigma$ is the standard deviation and $x$,$y$ are the image pixel locations. If the standard deviation $\sigma$ is too small, the off-center pixel weight will be very small, and the result is like there is no processing. If the standard deviation $\sigma$ is too large, the Gaussian template will degenerate into an average template. Actually, when the template is 3×3, $\sigma$ is 0.8. For a larger template, the value of $\sigma$ can be appropriately increased. The Gaussian template is shown in S-Tab. 1.

| | | 1/16 | 2/16 | 1/16 | | --- | --- | --- | | 2/16 | 4/16 | 2/16 | | 1/16 | 2/16 | 1/16 | | | --- | --- | --- | --- | --- | --- | --- | --- | --- | --- |     S-Tab. 1. The Gaussian template |
| --- | --- | --- | --- | --- | --- | --- | --- | --- | --- | --- |

**The Fast Otsu method**

The glottis is the central area of the throat. Segmenting the glottis is conducive to the subsequent vocal cord segmentation. The glottis belongs to the dark area in the image, so the grayscale value was used to segment the glottis. In order to highlight the difference of image brightness and retain more details, and avoid the result of over-segmentation, Fast Otsu method [9], which is less computational, was used to improve the disadvantage that the traditional Otsu method needs to calculate the inter-group variance of all grayscale values in order to obtain the best threshold value. The Fast Otsu method searches for the trend of the threshold value by calculating the variance between groups of the grayscale value squares, estimates the area where the optimal threshold value is located, and narrows the search region by iterating continuously, to find the grayscale value corresponding to the maximum intergroup variance, that is, the optimal threshold value.

The steps of the Fast Otsu method are as follows:

1. Calculating the average grayscale value of the entire image ($icp_{m}$), images above the average grayscale value are regarded as the foreground, and vice versa as the background, and calculating the average grayscale value of the two.

| $icp_{m}=\sum_{m=1}^{L} mp_{m}$ | (2) |
| --- | --- |
| $icp_{b}=\sum_{m=1}^{icp_{m}} mp_{m}$ | (3) |
| $icp_{f}=\sum_{m=icp_{m}}^{L} mp_{m}$ | (4) |

where$icp_{b}$is the average background value, $icp_{f}$is the average foreground value, $m$is the grayscale value 1~$L$, and$p_{m}$is the probability of the grayscale value $m$

2. According to step 1, the search area is divided into 0~$icp_{b}$, $icp_{b}$~$icp_{m}$, $icp_{m}$~$icp_{f}$ and $icp_{f}$ ~255. The inter-group variances of the four regions were calculated separately, and the largest inter-group variance was regarded as the possible optimal threshold.

3. Calculating the inter-group variances of the previous grayscale value and the next grayscale value of the possible optimal threshold value respectively. If the former value is the largest, the search direction proceeds to the previous region, and if the latter value is the largest, the search direction proceeds to the next region. If the middle value is the largest, the current threshold value is the optimal threshold value, and the iteration stops.

4. After the search direction is known by step 3, the new area $R_{a}$~$R_{b}$ is re-established, and the area is divided into 4 small equal parts, of which three dividing points$[cp_{m},cp_{b},cp_{f}]$of the 4 equal parts are calculated as follows.

| $cp_{m}=R_{a}+\frac{(R_{b}-R_{a})}{2}$ | (5) |
| --- | --- |
| $cp_{b}=R_{a}+\frac{(cp_{m}-R_{a})}{2}$ | (6) |
| $cp_{f}=cp_{b}+\frac{(R_{b}-cp_{m})}{2}$ | (7) |

Calculating the inter-group variances of the grayscale values of the three dividing points, regard the largest inter-group variance as the potential optimal threshold and return to step 3 for confirmation.

**ACM**

The vocal cords are blurred with the surrounding tissue boundaries. In this study, ACM [10] was used as the image segmentation method. The algorithm defines the initial parameterized curve, and makes the curve move to the target boundary by minimizing the energy function, i.e. the reconciliation of the internal and external forces of the image, and the boundary extraction is completed when the energy function reaches the minimum,

First, in the energy function, $c(r)=[x(r),y(r)]$ represents the parameter curve, $r\in[0,1]$, $x(r)$and $y(r)$represent the $x$and $y$ coordinates on the target contour, respectively. The image is represented by $c(r)=[x(r),y(r)]$, and the energy function is Eq. (8)

| $F(c)=\alpha\int_{a}^{b} \left\vert\frac{\partial c(r)}{\partial r}\right\vert^{2}dr+\beta\int_{a}^{b} \left\vert\frac{\partial^{2}c(r)}{\partial r^{2}}\right\vert+\int_{a}^{b} E_{ext}(c(r))dr$ | (8) |
| --- | --- |

where $\alpha$, $\beta$ are positive real numbers greater than 0, the first and second terms of the energy function are collectively referred to as internal energy (internal force) for the purpose of maintaining the continuity and stability of the curve, and the first one is theㄧorder differential term of the contour, which resists the tensile force mainly by controlling the slope of the contour; the second one is the second order differential term of the contour, which is used to resists the bending moment force.

The third term of the energy function is external energy, and $E_{ext}$ is defined as Eq. (9).

| $E_{ext}=\omega_{line}E_{line}+\omega_{edge}E_{edge}+\omega_{term}E_{term}$ | (9) |
| --- | --- |

The external force of the image is composed of three forces, $\omega$is the individual weight of the three forces. $E_{line}$ represents the line energy of the image , as Eq. (10)

| $E_{line}=-(G_{\sigma}*\nabla^{2}I_{0}^{2})$ | (10) |
| --- | --- |

where $G_{\sigma}$ is the Gaussian standard deviation, and $I_{0}$ is the image matrix.

$E_{edge}$ represents the edge energy of the image as Eq. (11)

| $E_{edge}=-\left\vert\nabla I_{0}(x,y)\right\vert^{2}$ | (11) |
| --- | --- |

$E_{term}$ denotes terminations energy. To obtain the termination condition, Kass et al. defined the curvature of the horizontal line on Gaussian blurred image. Let $C(x,y)$be the Gaussian blurred image $C(x,y)=G_{\sigma}(x,y)*I(x,y)$, define$\theta=\tan^{-1}(C_{y}/C_{x})$the gradient angle, and $n=(\cos\theta,\sin\theta)$ and $n_{\perp}=-(\sin\theta,\cos\theta)$represent the unit vectors horizontal and perpendicular to the gradient direction, respectively.

The horizontal contour curvature is shown as Eq. (12).

| $\begin{matrix} E_{term}=\frac{\partial\theta}{\partial n_{\perp}}=\frac{\partial^{2}C/\partial n_{\perp}^{2}}{\partial C/\partial n} \\ \text{ }=\frac{C_{yy}C_{x}-2C_{xy}C_{x}C_{y}+C_{xx}C_{y}^{2}}{(C_{x}^{2}+C_{y}^{2})^{3/2}} \end{matrix}$ | (12) |
| --- | --- |

The initial parameterized curve is mutually pulled by the internal and external forces of the energy function mentioned above, and finally the balance of the forces, that is, the minimized energy function is achieved, and the final contour boundary is obtained.

**Supplementary Figure 1. System image processing flow**

**
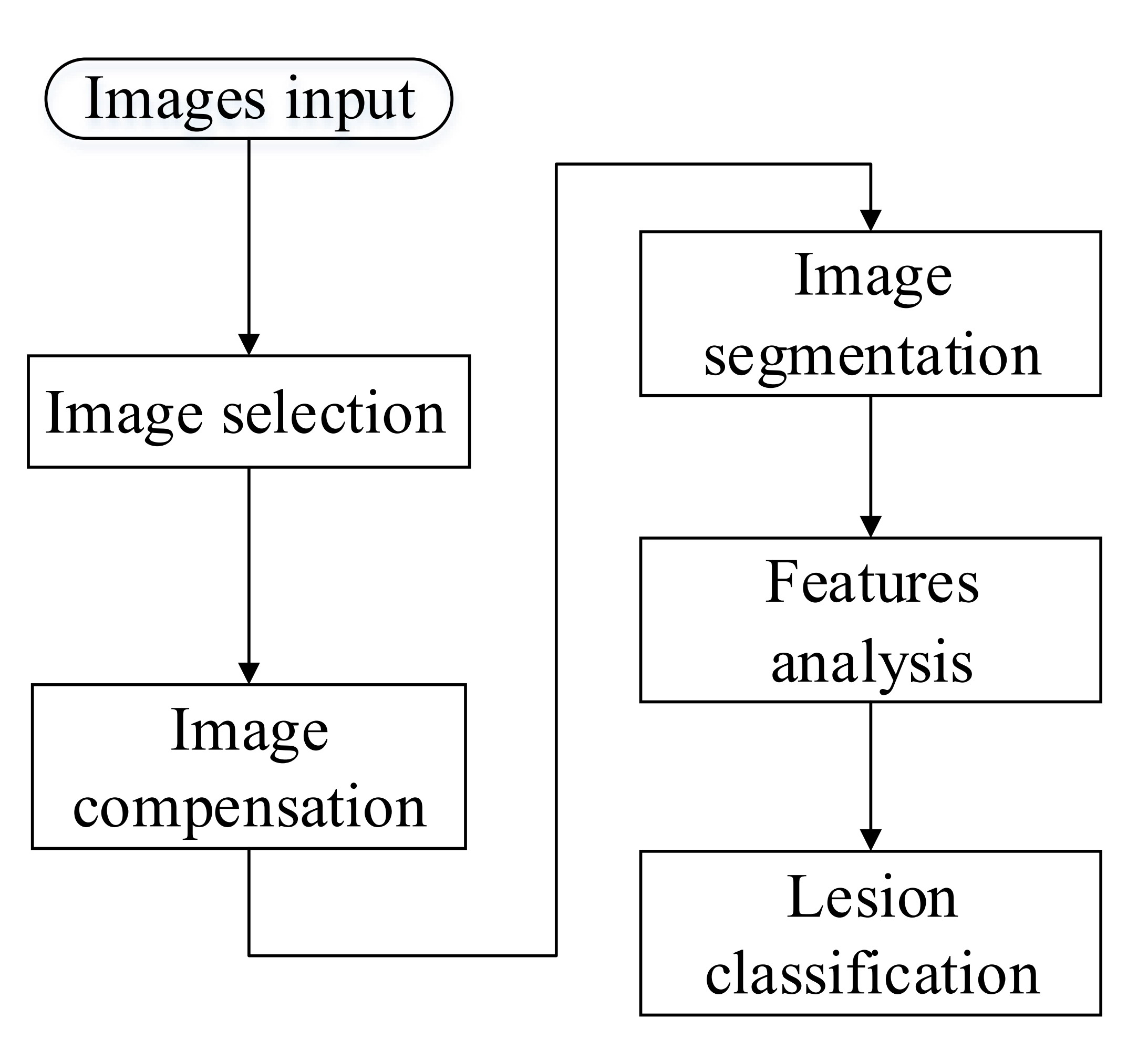
**

**Supplementary Figure 2.** Image shielding.

The peripheral areas in the original image (a) are black by the naked eye observation, but in computer analysis, these peripheral areas are composed of pixels with low-value grayscale pixels, which will interfere with subsequent segmentation and features analysis. In this study, the peripheral grayscale pixels were unified to 0 (b), so that the accurate values can be obtained by excluding the grayscale pixels of 0 from the subsequent calculations.

**
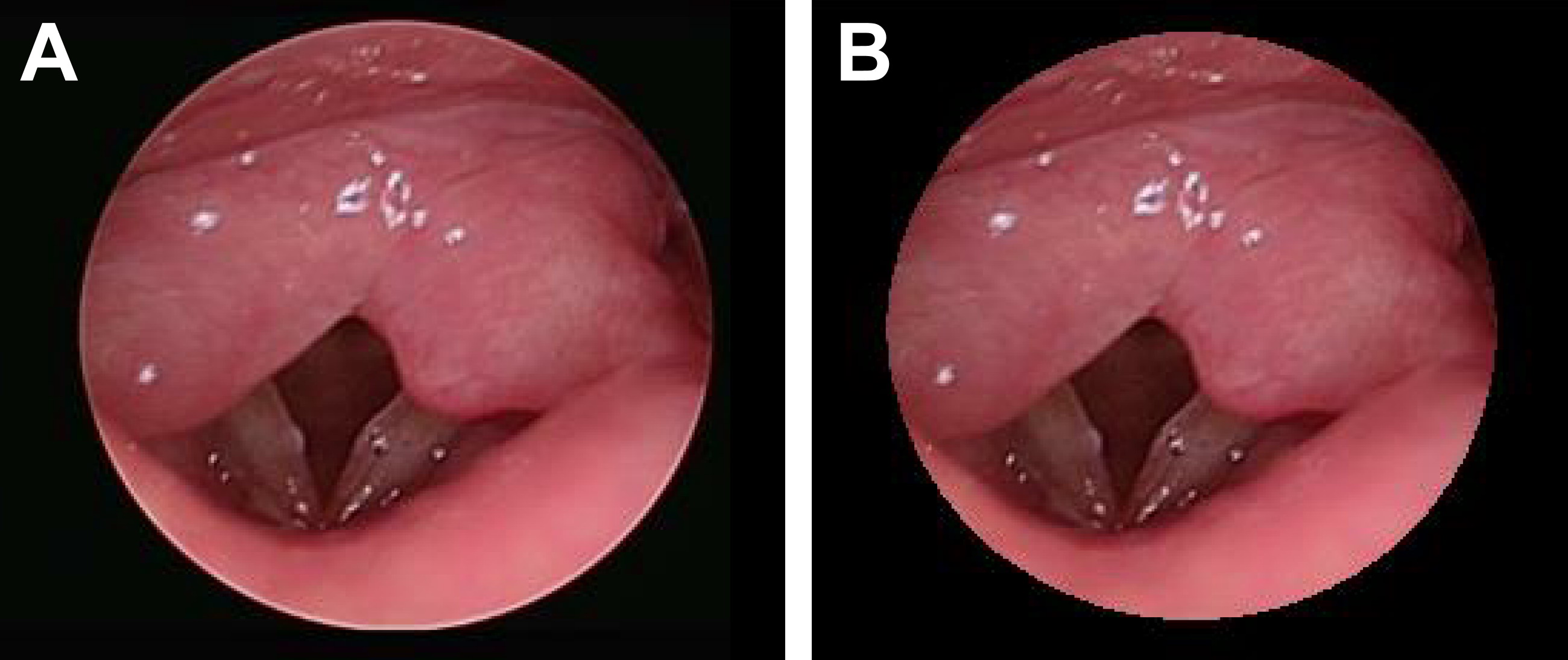
**

**Supplementary Figure 3.** Vocal cord seed point process. (a) Glottic image. (b) Glottic contour. (c) Vocal cords seed point.

**
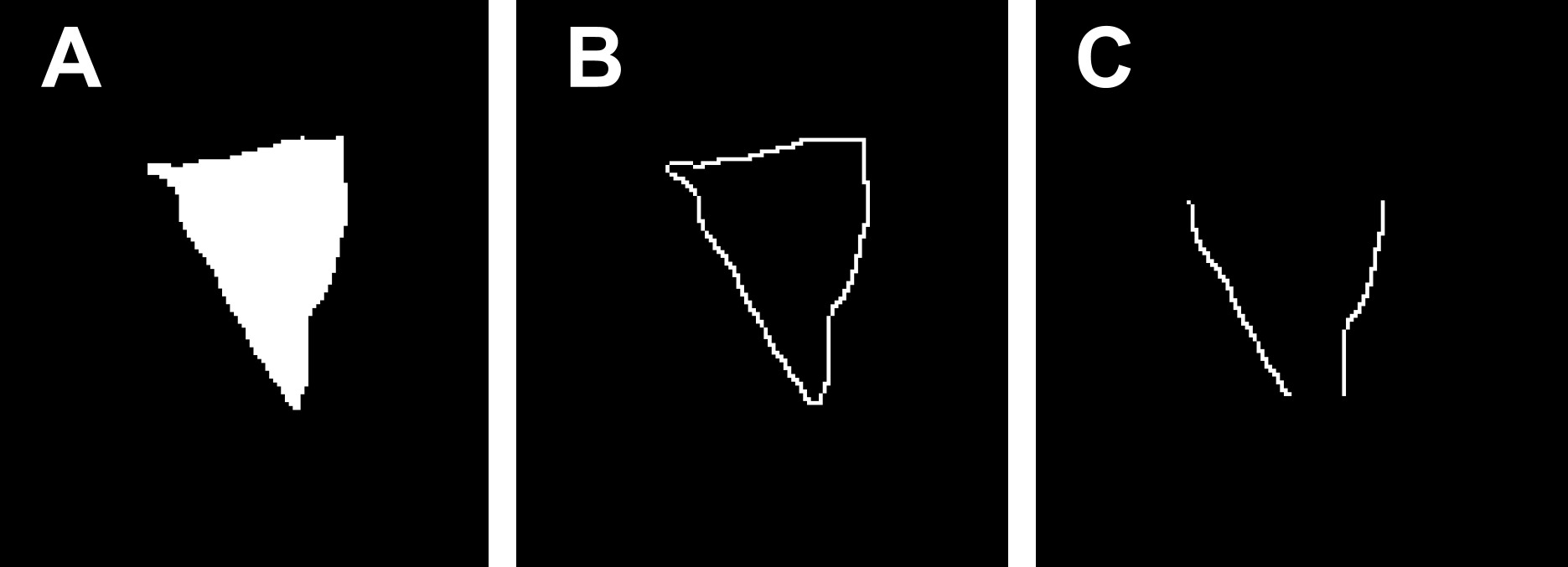
**

**Supplementary Figure 4. Vocal cord lesion features**

| The structure of the cyst is liquid and the shape is flat.  The structure of polyps is soft meat and the shape is convex.  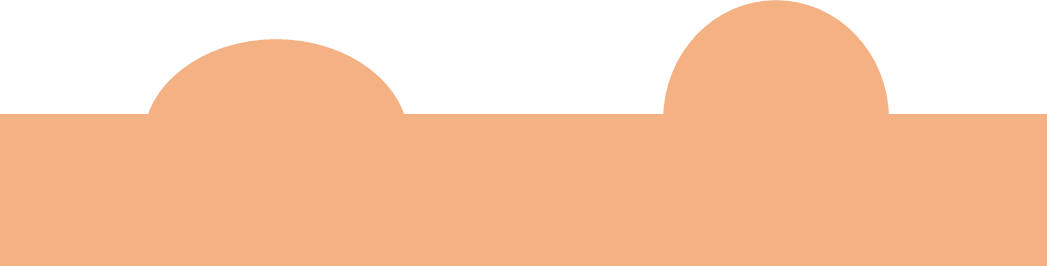 |
| --- |

Fig.5. Polyp and cyst shape difference
